# Resveratrol Regulates Antioxidant Status, Inhibits Cytokine Expression and Restricts Apoptosis in Carbon Tetrachloride Induced Rat Hepatic Injury

**DOI:** 10.1155/2011/703676

**Published:** 2011-10-15

**Authors:** Souvik Roy, Santanu Sannigrahi, Subhabrota Majumdar, Balaram Ghosh, Biswajit Sarkar

**Affiliations:** ^1^Department of Pharmaceutical Technology, NSHM Knowledge Campus, 124 B.L. Saha Road, Kolkata 700053, India; ^2^Department of Pharmacology, Calcutta Institute of Pharmaceutical Technology and AHS, Howrah, Uluberia 711316, India; ^3^Department of Pharmacology, Calcutta Medical College, West Bengal, Kolkata 700012, India

## Abstract

Recent studies indicate the chemopreventive role of resveratrol in many animal models like ischemia, rheumatoid arthritis, human cancer, and diabetes. The present study was designed to investigate the chemopreventive potential of resveratrol in rat hepatic injury model by carbon tetrachloride. Male Wistar rats were treated with carbon tetrachloride (0.4 g/kg body weight) intraperitoneally daily for 8 weeks. Resveratrol (100 mg/kg, 200 mg/kg body weight) was given orally from first day until the last day of experiment. The investigation assesses the effect of resveratrol on morphological, oxidative status, histopathological, immunohistochemical, and apoptotic analysis in carbon tetrachloride-challenged liver tissue. The study indicated that the inflammatory cytokines TNF-**α** and IL-6 were profoundly expressed in experimental rats, whereas resveratrol decreases the immunopositivity of TNF-**α** and IL-6 and restored the altered architectural structure of challenged hepatic tissue. Resveratrol also protects liver cells by suppressing oxidative stress and apoptosis.

## 1. Introduction

Liver is a major organ responsible for the metabolism of drugs and chemicals and also the primary target organ for many toxic chemicals. The environmental carcinogen, carbon tetrachloride (CCl_4_), has been considered as one of the best characterized experimental model for chemically induced rat liver damage. CCl_4_ is a classical hepatotoxicant causing liver cirrhosis, fibrosis, and necrosis by producing highly reactive trichloromethyl free radical, initiating lipid peroxidation and causing centrilobular necrosis. High levels of reactive oxygen species (ROS) induce cell damage and are involved in several human pathologies, including liver cirrhosis and fibrosis [1, 2]. Therefore, the use of compounds with antioxidant properties may prevent or alleviate many diseases associated with ROS. Formation of ROS is a naturally occurring process, and mammalian cells have developed several protective antioxidant defense mechanisms against its formation and detoxification [3]. ROS are also suggested to play an important role in cytochrome c release from mitochondria followed by apoptotic response. Damaging of kupffer cells, hepatic stellates, and sinusoid endothelial cells resulting in production of pro-inflammatory cytokines like IL-6, TNF-*α*, TGF-*β*, eNOS, which are intimately involved in chemical induced hepatotoxic process [4–7] causes infiltration of neutrophiles and monocytes into the damaged organ [8, 9]. TNF-*α*, IL-6 are considered as hepatotoxic in several experimental models of liver injuries [10, 11].

**Figure 1:**
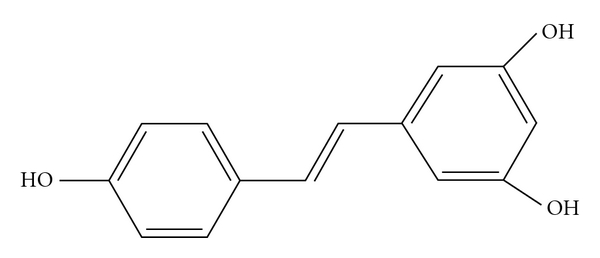
Structure of trans resveratrol.

Resveratrol (3,4′,5 trihydroxystilbene) is a naturally occurring polyphenol, mainly present in skin of grapes, red wine, mal berries, and peanuts (Figure 1). During the last few years most of the scientific evidence for resveratrol benefits is based on in vivo or in vitro studies in which this compound possesses several pharmacological activities including anticancer [12], antioxidant [13], anti-inflammatory [14], antidiabetic [15], antinociceptive, and antiasthmatic activity [16]. Reports also suggest that it has an effective role in glaucoma [17], pancreatitis [18], and osteoarthritis [19]. It prevents and cures cardiovascular diseases and improves microcirculatory disorders [20]. Resveratrol protects against oxidative stress in cholestasis and exerts hepatoprotective activity against ethanol- and thioacetamide-induced acute and chronic liver damage in rodents [21, 22]. Resveratrol at low doses decreases ischemia-reperfusion-induced liver injury and protects hepatocytes against antioxidant defense failure [23]. Resveratrol exhibits immunomodulatory effect by suppressing overproduction of inflammatory cytokines like TNF-*α*, IL-1*β*, and IL-6 [24].

Apoptosis is a genetically encoded form of cell suicide central to the development and homeostasis of multicellular organisms [25, 26]. Previous report suggested that increased hepatocyte apoptosis is an important mechanism contributing to inflammation and fibrogenesis of the liver [1]. There is a growing evidence to suggest that apoptosis is involved in rat liver damage at the early phase in CCl_4_-induced liver injury [27].

The events that trigger apoptosis in CCl_4_-induced acute hepatic injury are yet unknown. CCl_4_-induced toxicity in isolated hepatocytes is mediated by a direct solvent injury to cell membranes [28]. Nonparenchymal cells such as kupffer cells are activated by the release of cytokines, which may contribute to pathophysiological process culminating in hepatocyte apoptosis after toxic injury to the liver [29]. Therefore, we hypothesize that upon CCl_4_ insult, cytokines are expressed in the liver which alter the apoptotic process and may be reversed by the treatment of resveratrol.

Therefore the present study was undertaken to unveil the possible chemopreventive approach of resveratrol by examining the temporal changes of morphological, histopathological, and TNF-*α* and IL-6 immunoexpression correlating with the oxidative stress and apoptotic events in CCl_4_-induced subchronic liver damage of male Wistar rats.

## 2. Results

### 2.1. Body and Liver Weight

The mean body weights of different groups of rats are shown in Figure 3. No statistical differences were seen between the growth rates of any of the treatment-related groups and control group. A slight decrease of final body weight was observed in CCl_4_ control group (group B) as compared to normal control (group A), though this result was not statistically significant. Resveratrol-treated groups (group C & D) maintained the normal body weights compared to normal control (group A), and it suggested that resveratrol had practically no adverse effect on the rat growth response. The mean liver weight along with the relative liver weight of group B (*P* < 0.01) and group C (*P* < 0.05) is significant as compared to normal control. Whereas no statistical difference was observed in group D compared to normal control. Furthermore group D and group A showed significant difference (*P* < 0.01) as compared to CCl_4_ control (Table 1).

### 2.2. Effect of Resveratrol on CCl_4_-Induced Nodular Growth

Visible hepatocyte nodules were not observed in the livers of normal control (group A), whereas macroscopic nodules were clearly seen in the CCl_4_ control rat liver (Figure 4). Number of rats with nodules, incidence of nodules, total number of nodules, and nodular multiplicity of CCl_4_ control group along with resveratrol-treated groups were shown in Table 2. A significant (*P* < 0.05, *P* < 0.01) decreased incidence of nodules was observed in resveratrol-treated groups at a dose of 100 mg/kg (group C) and 200 mg/kg (group D) as compared to group B; the results were mostly prominent for group D. Likewise the total number of nodules was found to be less in all two resveratrol-treated groups as compared to group B. Nodular multiplicity was found to be significant (*P* < 0.01) in group C and D than group B. When a comparison was made between group C and D, a significance (*P* < 0.01) was emerged for the nodule multiplicity. 

Resveratrol treatment at a dose of 100 mg/kg (group C) and 200 mg/kg (group D) reduced the appearance of nodules and decreased the development of nodules of less than 3 mm, 1 mm in size when compared to group B (Table 3). Mean nodular volume was found to be significantly decreased (*P* < 0.05) in group C as compared to group B. Furthermore, statistically significant results were obtained (*P* < 0.01) in group D as compared to group B. Nodular volume as a percentage of liver volume was significantly decreased (*P* < 0.05) in group C in comparison with group B. A significant (*P* < 0.05, *P* < 0.01) decrease in nodular volume as a percentage of liver volume was observed in all resveratrol-treated groups as compared to group B. Group D also showed significant (*P* < 0.05) results when compared to group C. 

### 2.3. Effect of Resveratrol on Lipid Peroxidation, Hepatic GSH and GST

The effects of resveratrol on lipid peroxidation, hepatic GSH, and GST content in liver tissue were shown in Table 4. Malondialdehyde (MDA) is the end product and common marker of lipid peroxidation. The content of MDA was significantly (*P* < 0.01) higher in CCl_4_ control group. Treatment with resveratrol 100 mg/kg (group C) and 200 mg/kg (group D) significantly decreased the MDA content. As the oxidative stress of tissue generally involves the GSH and GST system, we measured the level of GSH and GST in each group of livers. The administration of CCl_4_ significantly depleted GSH and GST content; treatment with resveratrol significantly protected against the GSH and GST depletion induced by CCl_4_.

### 2.4. Effect of Resveratrol on Hepatic Histology

Identification and alteration of histopathological changes during hepatocellular damage and the protective effect of resveratrol against CCl_4_-induced toxicity were depicted in Figure 5. Histopathology of liver from the normal control (group A) showed a regular arrangement of hepatocytes with clearly visible nuclei (Figure 5(a)). Intraperitoneal injection of CCl_4_ at a dose of 0.4 g/kg body weight (group B) showed extensive centrilobular necrosis around the central vein of liver and dilation of the central vein with few regenerative areas of hepatocytes around the central vein, and distal part of liver was observed (Figure 5(b)). The magnitude of dilation of the central vein was prominent but very less in group C as compared with CCl_4_ control group (group B) (Figure 5(c)). The liver sections from group D showed good protection of hepatocellular necrosis with a poorly dilated central vein and regular arrangement of hepatocytes around the central vein. The distal part of the liver from the central vein was observed along with a scattered regenerative zone of hepatocytes; very few necrotic zones were prominent (Figure 5(d)). 

### 2.5. Effect of Resveratrol on Expression of TNF-*α*

The expression of TNF-*α* was analyzed by immunohistochemistry (Figure 6), and percentage of TNF-*α* immunopositive cells in liver tissues of various groups of experimental rats were shown in Table 5. The liver sections of normal control showed complete absence of immunostaining of TNF-*α* (Figure 6(a)). A large amount of TNF-*α* immunopositive cells (28.5 ± 2.4) were detected in the liver tissue sections of the CCl_4_ control rats (group B) (Figure 6(b)). In contrast, the decrement of TNF-*α* immunopositivity (15.3 ± 3.6), (*P* < 0.05) was observed upon resveratrol-(100 mg/kg) treated rats compared to CCl_4_ control (Figure 6(c)). Furthermore a more decrement of TNF-*α* immunopositivity was marked (10.6 ± 4.7, *P* < 0.01) in resveratrol-(200 mg/kg) treated rats compared to CCl_4_ control group (Figure 6(d)).

### 2.6. Effect of Resveratrol on Expression of IL-6

Figure 7 depicted the representative photomicrographs of immunohistochemical staining of IL-6 in liver tissues. Table 5 showed the expression of IL-6 immunopositive cells (as percentage) of various groups of experimental rats. The liver sections of normal control showed complete absence of immunostaining of IL-6 (Figure 7(a)). A large amount of IL-6 immunopositive cells (18.2 ± 0.7) were detected in the liver tissue sections of the CCl_4_ control rats (group B) (Figure 7(b)). In contrast, the decrement of IL-6 immunopositivity was observed (12.4 ± 0.3, *P* < 0.05) upon resveratrol (100 mg/kg) treatment compared to CCl_4_ control (Figure 7(c)). A more decrement of IL-6 immunopositivity was observed (8.2 ± 0.6, *P* < 0.01) in resveratrol-(200 mg/kg) treated rats compared to CCl_4_ control (Figure 7(d)).

### 2.7. Estimation of TNF-*α* and IL-6 by ELISA

Levels of cytokines (TNF-*α* and IL-6) in liver homogenate were shown in Figure 8. The levels of TNF-*α* (Figure 8(a)) and IL-6 (Figure 8(b)) were significantly (*P* < 0.01) elevated in CCl_4_ control group. Treatment with resveratrol at the dose 100 and 200 mg/kg significantly (*P* < 0.01) reduced the level of inflammatory cytokines. In addition with these, the effect of resveratrol at 200 mg/kg was found more significant (*P* < 0.01) when compared to resveratrol-100 mg/kg-treated group. 

### 2.8. Effect of Resveratrol on Apoptosis

The CCl_4_ injection caused necrosis along with apoptosis in a dose- and duration dependent-manner in experimental animal models. The immunohistochemical analysis of apoptosis was performed by TUNEL method (Figure 9). The chromogen-generated brown stain is an indication of apoptotic cells. It should be noted that in almost every case, the brown stain overlaps the condensed chromatin of apoptotic bodies, thus confirming that the TUNEL assay correlates well with the morphological appearance of apoptosis. The rate of apoptosis was generally very low in normal control group (Figure 9(a)), and more positive staining was expressed in CCl_4_-induced liver sections (Figure 9(b)). Less apoptotic cells were observed in resveratrol-treated groups (group C and D) (Figures 9(c) and 9(d)). The percentage of TUNEL positive apoptotic cells was denoted as apoptotic index. The apoptotic index value of group B was found as 21.3 ± 1.2. Reduction of apoptosis (13.71 ± 0.17) by resveratrol (100 mg/kg) was statistically significant (*P* < 0.05) as compared to group B. Furthermore, the reduction of apoptosis (10.6 ± 0.28) in high dose of resveratrol (200 mg/kg) was more statistically significant (*P* < 0.01) when compared to CCl_4_ control group. The apoptotic index value of group D was significantly less (*P* < 0.05) as compared to group C.

## 3. Discussion

The antioxidant and free radical scavenging activities of many substances have been assessed, and reports suggested that compounds having strong antioxidant activity showed promising hepatoprotective activity [30, 31]. Polyphenolic compounds, which are widely distributed in fruits, are considered to play an important role as dietary antioxidants for the prevention of oxidative damage during pathogenesis [32]. Therefore, we hypothesized that resveratrol, a polyphenolic compound, would be beneficial in the prevention of various liver injuries induced by oxidative stress. 

Liver injury induced by CCl_4_ is the best characterized system of xenobiotic induced hepatotoxicity and is a commonly used model for screening hepatoprotective drugs [33–35]. Earlier studies described the effect of resveratrol on acute liver damage induced by high dose of CCl_4_ [36]. Resveratrol was found to prevent fibrosis by reduction of NF-kappa*β* activation and TGF-*β* content in CCl_4_ induced liver damage in rats [37]. Our data first indicates that subchronic CCl_4_ treatment provoked a clear toxicity in rats, as assessed by morphological variations reflected by markedly increased and localized in hepatic nodules, alteration of oxidative status and histopathological architecture of hepatic tissue, and overexpression of proinflammatory cytokines. Till now, the chemopreventive studies of resveratrol against CCl_4_-induced subchronic toxicity and its relation with apoptosis and levels of inflammatory cytokines have not been reported. In present study it has been recognized that CCl4-induced hepatotoxicity is mediated through an apoptotic mode of action of hepatocytes secondary to the cytolethality. Resveratrol appears as a good candidate in the prevention of CCl4-induced rat liver injury. In our present study, the body weight of experimental animals was not altered, and this may be considered to be an important aspect of resveratrol action.

The findings of the present investigation demonstrated that resveratrol treatment greatly reduced CCl_4_-initiated hepatic damage in rats, and administration of resveratrol by oral route ameliorates nodule formation. Even though during the life span of animals not all the hepatocyte nodules become malignant but numerous studies support that the neoplastic/hyperplastic nodules are the precursors of hepatic cancer. In present study, the effect of resveratrol on inhibition of nodule growth and enhancement of their regression was observed.

Previous studies suggested that GSH plays a key role in detoxifying the reactive toxic metabolites of CCl_4_. Failure to detoxify the metabolites promotes liver necrosis [38, 39]. GSH form adducts with the toxic metabolites of CCl_4_ and contributes to the detoxification of CCl_4_. It has been suggested that one of the principal causes of CCl_4_-induced liver injury is lipid peroxidation caused by its free radical derivatives [38]. GST is a soluble protein located in the cytosol that plays an important role in the detoxification and excretion of xenobiotics [40]. Moreover, GST functionally binds with GSH and endogenous and exogenous substances. Our findings showed that treatment with resveratrol significantly inhibited lipid peroxidation, reduced CCl_4_-induced hepatic GSH depletion, and enhanced the GST activity.

Histological findings clearly showed that the normal architecture of the hepatic tissue was altered due to administration of CCl_4_-released reactive oxygen species causing degeneration in hepatocytes, extensive centrilobular necrosis around the central vein of liver. In contrast, rats treated with resveratrol showed noticeable improvement in histopathological parameters. There was no sign of necrotic region and dilation of central vein. Our result is also at per of recent findings that resveratrol protects primary rat hepatocytes against cell damage induced by reactive oxygen species [41].

In this study, we analyzed the inhibitory effect of resveratrol on the release of proinflammatory cytokines in CCl_4_-induced rat hepatic damage model. Cytokines serve as central regulators controlling genes, responsible for either inducing apoptosis or protective action on cell by stimulating proliferation of hepatocyte. Cytokines constitute a complex network involved in the regulation of inflammatory responses and maintain homeostasis of organ functions. Major hepatotoxic mediators in several experimental models of liver injury are TNF-*α* and IL-6. TNF-*α* is a circulating factor which causes necrosis of tumors when administered to tumor-bearing mice. These levels are elevated in both infiltrating inflammatory cells and hepatocytes in chronic liver injuries including viral or alcoholic liver diseases, hepatitis, ischemia, and biliary obstruction [42]. Exposure to hepatotoxic chemicals facilitates TNF-*α* to induce necrotic cell death and hepatic apoptosis [43]. IL-6 is a multifunctional cytokine along with TNF-*α* activates hepatic stellate cells and increases the production of extracellular matrix proteins that finally leads to cirrhosis. Elevation of IL-6 concentration after hepatic injury suggested a potential role of IL-6 in hepatocyte proliferation, but some studies demonstrated that hepatocyte regeneration was impaired in IL-6 deficient mice [44]. In our recent findings, overexpression of TNF-*α* and IL-6 in CCl_4_ control rats was markedly reduced by resveratrol-treated groups.

It was well documented that necrosis in the centrilobular zone is beloved to the injury of hepatocytes. However both necrosis and apoptosis can be caused by drugs and chemicals. It was studied that apoptosis is preprogrammed form of cell death which is characterized by organized cellular fragmentation; remnants resulting from this structured decay, termed apoptotic bodies, are then cleared by phagocytosis. Pathological rates of unregulated apoptosis associated with an enhanced inflammatory response and fibrosis. We investigated the possible role of apoptosis in the carbon tetrachloride-induced liver injury and its relation with chemopreventive nature of resveratrol. Our results indicate that cell loss by apoptosis occurred profoundly and rapidly in the carbon tetrachloride-challenged liver and expressed more number of TUNEL positive cells. Suppression of apoptotic incident was observed by resveratrol-treated groups.

In conclusion the protective effect of resveratrol was confirmed in a well-defined rat model of carbon tetrachloride-induced liver injury. Recently much attention is focused on the protective functions of naturally occurring antioxidants in biological systems and their mechanisms. This study provides biological evidence that supports the use of resveratrol in the treatment of liver disorder. Downregulation of apoptosis and suppression of inflammatory cytokines levels can be attributed to molecular mechanism of hepatoprotective activity of resveratrol. Considerable efforts are being undertaken to develop specific and safe biological and molecular inhibitors of TNF-*α* and IL-6 for potential therapeutic use. The understanding of the molecular basis for inflammatory diseases to improve human health risk assessment and also identification of functional polymorphisms for inflammatory mediators should allow for a better estimation of determining susceptible populations and help to improve human risk assessment.

## 4. Materials and Methods

### 4.1. Chemicals

Resveratrol, carbon tetrachloride (CCl_4_), thiobarbituric acid reagent (TBA), reduced glutathione (GSH), biotinylated rabbit anti-mouse IgG, streptavidin peroxidase, antidigoxigenin peroxidase, 3,3 diaminobenzidine tetrahydro chloride (DAB), terminal deoxynucleotidyl transferase (TdT), and deoxyuridine triphosphate (dUTP) were obtained from Sigma Chemical Company (St. Louis, Mo, USA). ELISA kit was purchased from BD Bioscience (San Jose, Calif, USA). Glutathione-S-transferase detection kit was purchased from Stratagene (La jolla, Calif, USA). The rabbit anti-mouse TNF-*α* and IL-6 antibody procured from Anaspec Inc. (San Jose, Calif, USA). All other chemicals and reagents were used of analytical grade purchased purest form available from local firms.

### 4.2. Animals and Diet

Pathogen-free male Wistar rats, initially weighing 100–150 g, were obtained from Indian Institute of Chemical Biology (IICB, Kolkata, India). The animals were acclimatized to standard laboratory conditions (temperature 18C ± 2C, relative humidity 40% to 70% and a 12 h : 12 h light/dark cycle) and housed in solid bottom polypropylene cages (four animals per cage) bedding for one week before starting the experiment. The animals were fed with a semipurified basal diet and demineralized drinking water *ad libitum*. The recommendations of “Institutional Animal Ethical Committee” [Committee for the Purpose of Control and Supervision of Experiments on Animals (CPCSEA Regn. no. 1126/bc/07CPCSEA) India] for the care and use of laboratory animals were strictly followed throughout the experiment.

### 4.3. Induction of Hepatic Injury

The experimental hepatic injury was initiated by intraperitoneal injection of carbon tetrachloride (0.4 g/kg/day) dissolved in purified mineral oil and continued up to 8 weeks. It is an important xenobiotic swallowing or inhalation exposure of CCl_4_ over a period of time which increases the frequency of liver damage in animals [45].

### 4.4. Resveratrol Dose Selection

The doses of resveratrol 100 mg and 200 mg/kg bodyweight/day were selected based on previously reported chemopreventive and toxicological properties of this compound in rats. These doses have been found to suppress diethyl nitrosamine-initiated hepatocarcinogenesis in rodent model [46]. Resveratrol is not soluble in water therefore administered by suspended in 0.7% of carboxymethyl cellulose.

### 4.5. Experimental Design

**Figure 2:**
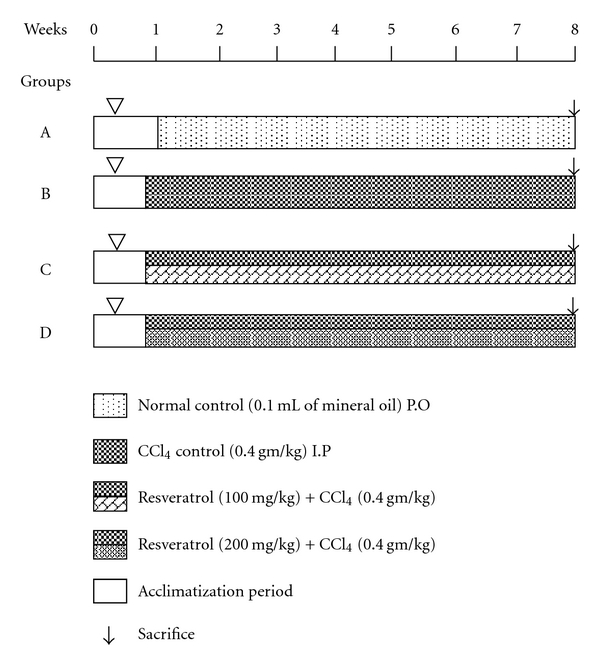
Schematic representation of experimental regimen.

**Figure 3:**
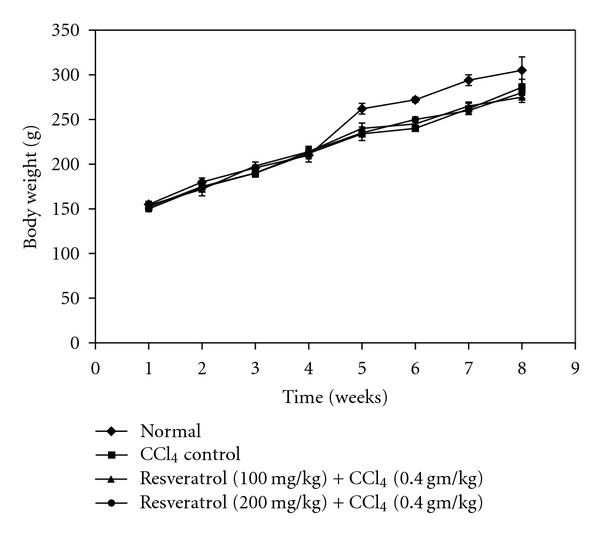
Effect of resveratrol on body weight gain during hepatic damage in rats. No significant difference in body weight was detected among the four groups.

**Figure 4:**
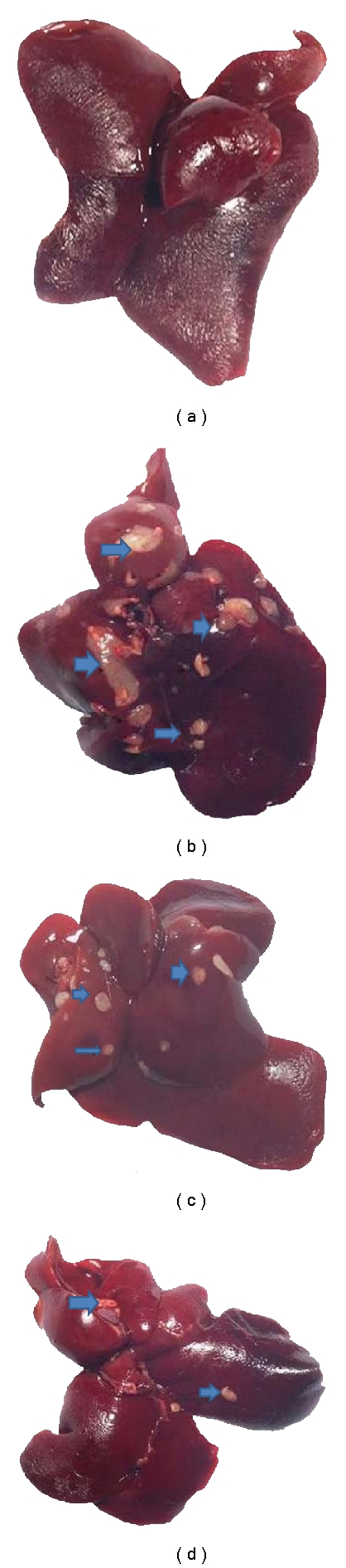
Morphological examination of rat liver tissue at the end of the study. Macroscopically visible hepatic nodules were shown by arrows. Representative livers are taken from several groups: (a) Normal control (group A); (b) CCl_4_ control (group B); (c) Resveratrol 100 mg/kg + CCl_4_ (0.4 g/kg) (group C); (d) Resveratrol 200 mg/kg + CCl_4_ (0.4 g/kg) (group D).

**Figure 5:**
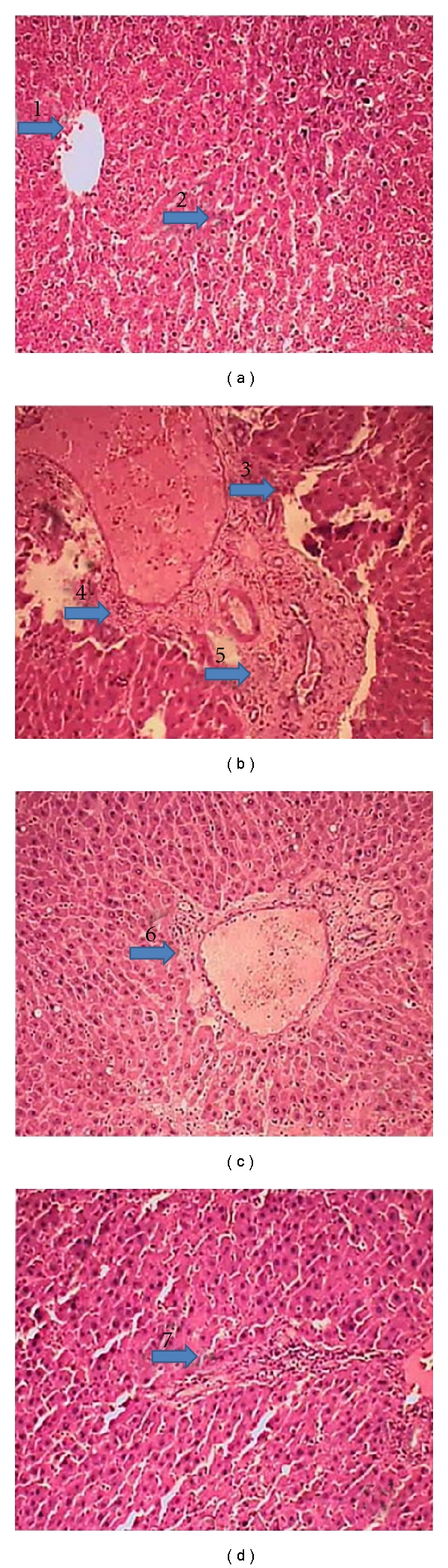
Histological profile of representative liver tissue in experimental animals. (a) Normal control rat liver (group A). Arrow 1 indicates the normal structure of central vein with radially arranged hepatocytes around it. Arrow 2 indicates normal hepatocytes (H & E X 200); (b) CCl_4_ control (group B). Arrow 3 showed dilation of the central vein with few regenerative areas of hepatocytes around the central vein and distal part of liver. 4, 5 arrow showed extensive centrilobular necrosis around the central vein of liver (H & E X 200); (c) section from resveratrol (100 mg/kg) + CCl_4_ (group C) arrow 6 represents the magnitude of dilation of the central vein (H & E X 200); (d) section from resveratrol (200 mg/kg) + CCl_4_ ( group D) arrow 7 showed good protection of hepatocellular necrosis with a poorly dilated central vein and regular arrangement of hepatocytes around the central vein and also the distal part of the liver from the central vein along with a scattered regenerative zone of hepatocytes; very few necrotic zones were prominent showing hepatocytes maintaining nearnormal architecture (H & E X 200).

**Figure 6:**
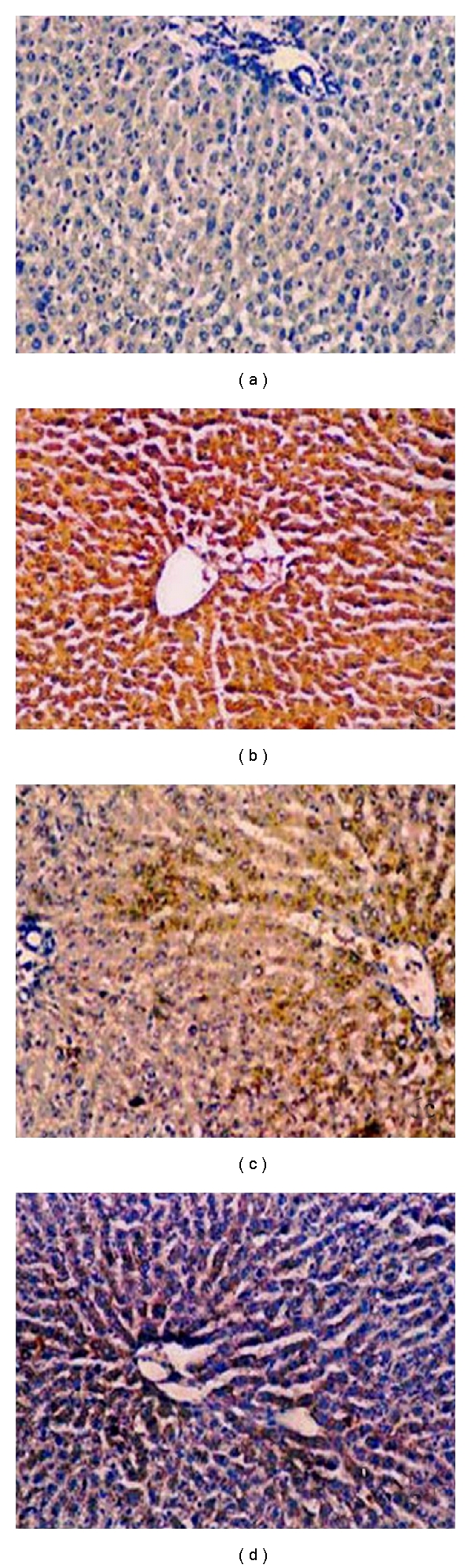
Immunohistochemical localisation of TNF-*α* in the rat liver tissue (H & E X 100). The figure shows representative staining for TNF-*α* (DAB) against counterstain (hematoxylin). (a) Represents normal liver (group A); (b) represents CCl_4_ control liver (group B); (c) represents resveratrol (100 mg/kg) + CCl_4_ (group C); (d) represents section from resveratrol (200 mg/kg) + CCl_4_ (group D). The liver sections of control animals are mainly negative. Liver sections of CCl_4_-exposed rats show more intensive staining.

**Figure 7:**
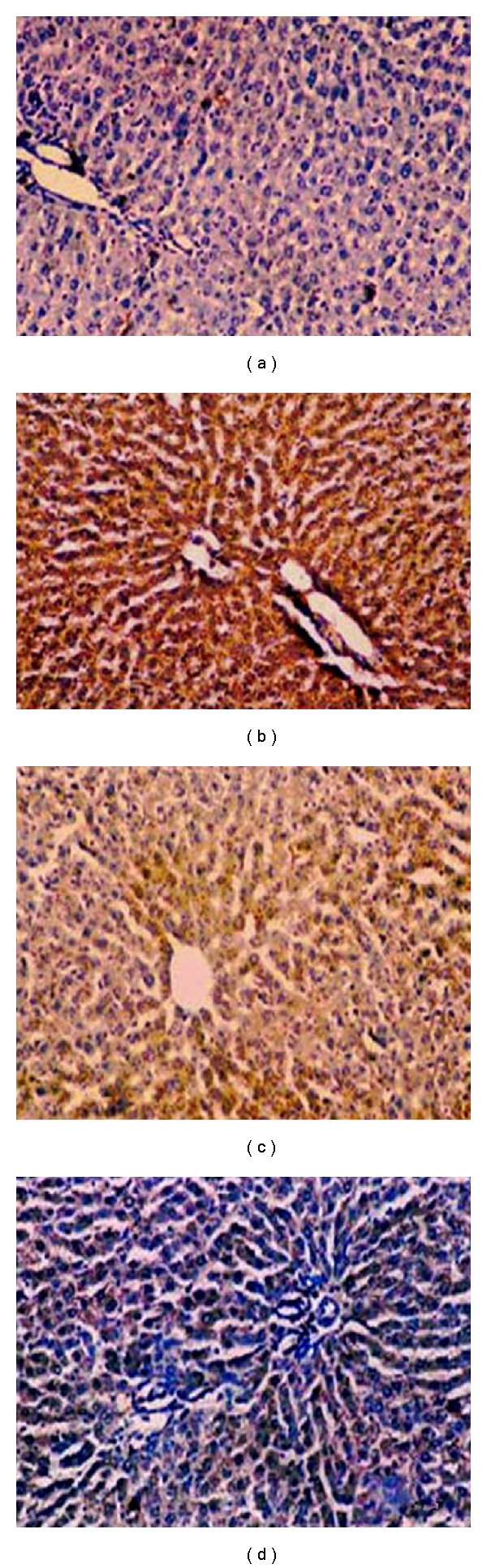
Immunohistochemical localisation of IL-6 in the rat liver tissue (H & E X 100). The figure shows representative staining for TNF-*α* (DAB) against counterstain (hematoxylin). (a) Represents normal liver (group A); (b) represents CCl_4_ control liver (group B); (c) represents resveratrol (100 mg/kg) + CCl_4_ (group C); (d) represents section from resveratrol (200 mg/kg) + CCl_4_ (group D). The liver sections of control animals are mainly negative. Liver sections of CCl_4_ exposed rats show more intensive staining.

**Figure 8:**
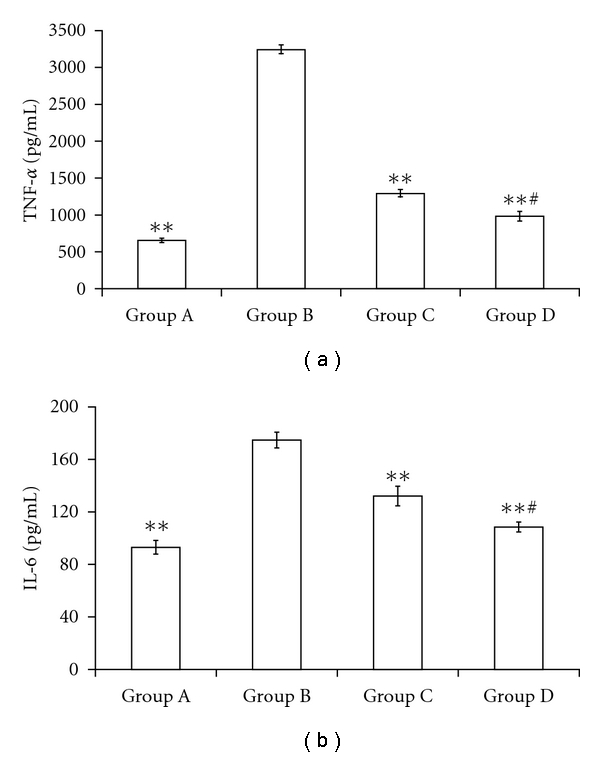
Levels of inflammatory cytokines TNF-*α* (a), and IL-6 (b) by ELISA in liver homogenate. Group A—Normal control; Group B—CCl_4_ control; Group C—resveratrol (100 mg/kg) + CCl_4_; Group D—resveratrol (200 mg/kg) + CCl_4_. The values are expressed as mean ± SEM (*n* = 10); *P < 0.01 as compared with Group B; ^#^ *P* < 0.01 as compared to group C.

**Figure 9:**
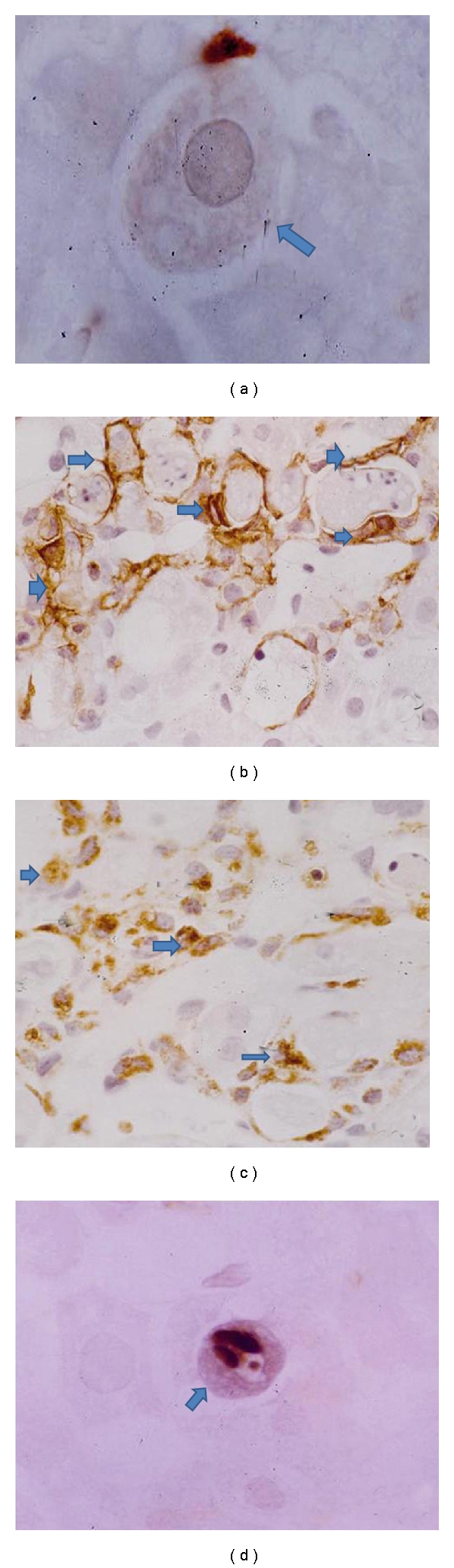
Apoptotic assay was performed by using TUNEL assay (H.E. X 400); brown stained cells (indicated by arrows) are undergoing apoptosis. (a) Immunostaining of rat liver from normal control (group A) showed absent of brown staining, (b) CCl_4_ control (group B) liver showing more staining indicative cells undergoing apoptosis; (c) resveratrol (100 mg/kg) + CCl_4_ (group C) exhibited less cells undergoing apoptosis; (d) resveratrol (200 mg/kg) + CCl_4_ (group D) exhibited very few number of brown-stained nuclei.

After acclimatization, rats were divided randomly into four groups with ten animals each (Figure 2). Normal control (group A) was treated with 0.1 mL of mineral oil and CCl_4_ control (group B) was treated with CCl_4_ (0.4 g/kg/day). Treatment group (group C) animals received resveratrol at a concentration of 100 mg/kg orally along with CCl_4_ (0.4 g/kg/day) and continued up to 8 weeks. Another group (group D) was treated with resveratrol at a concentration of 200 mg/kg orally along with CCl_4_ (0.4 g/kg/day) and continued up to 8 weeks. Body weights of animals were recorded every one week. After 8 weeks, animals were kept for overnight fasting and sacrificed randomly. Liver sections were collected and used for further experiment.

### 4.6. Morphology

Animals from each group were sacrificed with ether anesthesia. The livers were quickly excised, rinsed with ice-cold phosphate-buffered saline (pH 7.4) to flush out any blood, blotted dry on a paper towel, weighed, and photographed. Each liver was examined macroscopically on the surface as well as in 3 mm cross-sections for gross visible hepatocyte nodules from all groups of the rats. The nodules were measured with a vernier caliper through its two perpendicular planes to the nearest mm to obtain an average diameter of each nodule. The nodules were counted and categorized into two groups according to their respective diameters (<3–>1 and <1 mm). Nodular volume (*V*) was determined by using the formula

(1) $$V=\frac{4}{3}\pi r^{3},$$

where *r* = half of the average diameter (mm). 

### 4.7. Assessment of Lipid Peroxidation

Hepatic lipid peroxidation levels were determined by measuring thiobarbituric acid-reactive substance (TBARS) [47]. Samples were mixed with thiobarbituric acid (TBA) reagent consisting of 0.375% TBA and 15% trichloroacetic acid in 0.25 N hydrochloric acid. The reaction mixtures were placed in a boiling water bath for 30 min. The samples were centrifuged (2000 rpm for 10 min at 48°C), and the TBARS concentration was determined based on the absorbance at 532 nm measured with a spectrophotometer at room temperature.

### 4.8. Hepatic GSH and GST Activity

Livers were quickly removed and weighed and perfused with ice-cold 0.15 M KCl and then homogenized with 4 volumes of 10 mM *tris-*HCl (pH 7.4) containing 0.15 M KCl, 0.1 mM EDTA, 1.0 mM dithiothreitol, and 0.01 mM phenylmethylsulfonyl fluoride in an Ultra Turrax tissue homogenizer. Hepatic GSH levels were estimated colorimetrically (Systronic 2202, Ahmedabad, India) using Ellman's reagent [48]. Cytosolic GST activity was determined using 1-chloro-2,4-dinitrobenzene as a substrate [49].

### 4.9. Histopathology

Eight weeks after the CCl_4_ or vehicle treatment animals from each group were randomly selected; a portion of the liver was excised from ether-anaesthetized rat fixed in 10% formalin and processed for histopathological studies. The tissues were dehydrated through 70, 90, and 100% alcohol and embedded in low melting point paraffin wax. Section of 5 *μ*M thickness was cut and placed serially on glass slide. Each liver tissue was stained with hematoxylin and eosin for histopathological evaluation using light microscopy. 

### 4.10. Immunostaining of TNF-*α*, IL-6

Immunohistochemical detection of TNF-*α* and IL-6 in cold acetone fixed, paraffin-embedded liver sections was performed by the avidin-biotin-peroxidase complex method. Briefly 5 *μ*M thin sections of lysine coated slides were deparaffinised and rehydrated. For immunolabeling of TNF-*α* and IL-6 antigen retrieval was facilitated by heating the sections in citrate buffer pH 6.0, for 20 min. Endogenous peroxidase activity was blocked with 1% H_2_O_2 _ in 0.1 M Tris-NaCl, (pH 7.6) for 30 min. After incubated in 5% normal goat serum, sections were then separately incubated overnight at 4°C with the primary purified anti-mouse TNF-*α* antibody, primary purified anti-mouse IL-6 antibody at 1 : 50 dilutions, respectively, in 1% bovine serum albumin (BSA). Sections were then incubated with a biotinylated secondary antibody, goat anti-rabbit IgG (Sigma) for 30 min at 37°C with 1 : 100 dilutions. This was followed by incubation with streptavidin peroxidase (1 : 100) for 1 h. After that 100–400 *μ*L of diaminobenzidine tetrahydro chloride (DAB) reagent was added to each section. As soon as sections turn brown, the slides are immersed in double-distilled water (ddH_2_O) two times for 5 min each. The sections were dehydrated and mounted with DPX and served as a positive control. The percentage of immunopositive cells was counted under a light microscope.

### 4.11. Enzyme-Linked Immunosorbent Assays (ELISA)

Hepatic TNF-*α* and IL-6 levels were estimated using commercial ELISA kit purchased from BD Biosciences (San Jose, Calif) as per the protocol provided by the manufacturer. Microplates were coated with 100 *μ*L/well of capture antibody and incubated overnight at 4°C. After washes, the plates were blocked with assay diluent (BD Biosciences) at room temperature (RT) for 1 h. One hundred microliters of liver homogenate in PBS supplemented with protease inhibitors, were added to each well of the plate, followed by incubation for 2 h at RT. Working detector (100 *μ*L) was loaded into each well, and the plate was incubated for an additional 1 h at RT before the addition of 100 *μ*L substrate solution. The reaction was stopped by adding 50 *μ*L stop solution. The absorbance was read at 450 nm, with reference wavelength at 570 nm using a 96-well plate spectrometer (SpectraMax 190; Sunnyvale, Calif). Calculation of the concentrations of the cytokines was performed in a log-log linear regression according to the instructions in the protocol.

### 4.12. Apoptosis by TUNEL Assay

Apoptotic cells in liver tissue sections were determined by TUNEL assay. The sections were digested with proteinase K (20 *μ*g/mL in PBS) for 15 min at room temperature and rinsed with double-distilled water. Slides were then quenched by 2% H_2_O_2_ for 5 min at room temperature, equilibrated with buffer. Slides were then incubated with terminal deoxynucleotidyl transferase (TdT) buffer (30 mM Trizma base, pH 7.2, 140 mM sodium cacodylate, 1 mM cobalt chloride), followed by TdT reaction solution containing TdT and deoxyuridine triphosphate (dUTP) for 90 min at 37°C, then washed with 2% standard saline citrate to stop the reaction for 10 min at room temperature. The slides were then washed with PBS for 5 min each and incubated with antidigoxigenin peroxidase for 30 min (1 : 100) at room temperature. Color was developed using 0.05% of diaminobenzidine tetrahydro chloride (DAB Kit Sigma, St. Louis, Mo, USA) in 0.01% H_2_O_2_ diluted with *tris*-HCl (pH 7.5), then lightly counterstained with leishman stain. Sections were then washed, dehydrated, and mounted. Apoptotic cells were identified by a brown stain over the nuclei. Approximately 200 cells were counted per field, five fields were examined per slide, and five slides were examined per group. Apoptotic index (AI) was determined as the percentage of labelled cells (TUNEL positive) with respect to the total number of cells counted using (see Table 6)

(2) $$\begin{array}{cc} & \text{Apoptotic}\;\text{index}\;\left(\text{AI}\right)\\ & \;\;=\left(\frac{\text{number}\;\text{of}\;\text{labeled}\;\text{cells}}{\text{total}\;\text{number}\;\text{of}\;\text{cells}\;\text{counted}\;}\right)\times 100. \end{array}$$

### 4.13. Statistical Analysis

The data are expressed as means ± S.E.M. Comparisons are carried out by One way ANOVA followed by posttest (Dunnett's test) as appropriate using graph pad prism. Results were considered statistically significant, when *P* < 0.01, *P* < 0.05.

## Figures and Tables

**Table 1:** Liver weight and relative liver weight of different groups of rats at the end of study (after 8 weeks).

Groups	No. of rats	Liver weight (g)	Relative liver weight (g liver/100 g body weight)
A	10	10.5 ± 0.63^#^	3.62 ± 0.45^#^
B	10	13.0 ± 1.6**	4.67 ± 0.7**
C	10	12.2 ± 1.9*	4.51 ± 0.35*
D	10	11.0 ± 1.2^#^	3.88 ± 0.4^#^

Group A, normal control; group B, CCl_4_ administered rats; group C, resveratrol-(100 mg/kg) treated rats; group D, resveratrol-(200 mg/kg) treated rats.

Results are mean ± S.E.M for 10 animals; One way ANOVA followed by Dunnett's multiple comparison test.

*indicates value significantly different from the normal control at *P* < 0.05.

**indicates value significantly different from the normal control at *P* < 0.01.

^#^indicates value significantly different from the CCl_4_ control at *P* < 0.01.

**Table 2:** Effect of resveratrol treatment on the development of microscopic hepatocyte nodules induced by CCl_4_ in male Wistar rats.

Groups	No. of rats with nodules/total rats	Nodule incidence (%)	Total no. of nodules	Average no. of nodules/nodule-bearing liver (nodule multiplicity)
B	10/10	100	246	24.6 ± 2.4
C	6/10	60*	65	10.8 ± 1.3**
D	3/10	30**	21	7 ± 1.6^∗∗#^

Group B, CCl_4_-administered rats; group C, resveratrol-(100 mg/kg) treated rats; group D, resveratrol-(200 mg/kg) treated rats.

Results are mean ± S.E.M for 10 animals; One way ANOVA followed by Dunnett's multiple comparison test.

*P < 0.05 and **P < 0.01 as compared with group B.

^#^ *P* < 0.05 as compared with group C.

Animals from group A did not show visible hepatocyte nodules.

**Table 3:** Effect of resveratrol on the size distribution and growth of hepatocyte nodules induced by CCl_4_ in rats.

Groups	No. of rats with nodules	Nodules relative to size (% of total no.)		Mean nodular volume (cm^3^)	Nodular volume/liver volume (%)
		<3–>1 mm	<1 mm		
B	10	59 ± 7	41 ± 14	0.17 ± 0.04	1.3 ± 0.34
C	6	49 ± 15	51 ± 12	0.06 ± 0.02*	0.49 ± 0.14*
D	3	31 ± 12	69 ± 7	0.03 ± 0.05**	0.30 ± 0.22^∗∗#^

Group B, CCl_4_-administered rats; group C, resveratrol-(100 mg/kg) treated rats; group D, resveratrol-(200 mg/kg) treated rats.

Results are mean ± S.E.M for 10 animals; One way ANOVA followed by Dunnett's multiple comparison test.

*P < 0.05 and **P < 0.01 as compared with group B.

^#^ *P* < 0.05 as compared with group C.

Animals from group A did not show any visible hepatocyte nodule.

**Table 4:** Effect of the resveratrol treatment (100 mg/kg, 200 mg/kg body weight) for 8 weeks on the lipid peroxidation, glutathione reductase, and glutathione-s-transferase in liver tissue of CCl_4_-induced rats.

Group	TBARS (nmol/g liver weight)	GSH content (nmol/mg protein)	GST content (nmole/min/mg protein)
A	106 ± 2.4**	110 ± 2.2**	1622 ± 3.2**
B	303 ± 3.2	75 ± 1.5	720 ± 2.5
C	210 ± 3.5*	90 ± 2.2*	1145 ± 2.4*
D	129 ± 2.7^∗∗#^	107 ± 1.2^∗∗#^	1543 ± 3.6^∗∗#^

Group A, normal control; group B, CCl_4_-administered rats; group C, resveratrol-(100 mg/kg) treated rats; group D, resveratrol-(200 mg/kg) treated rats.

Results are mean ± S.E.M for 10 animals; One way ANOVA followed by Dunnett's multiple comparison test.

*P < 0.05 and **P < 0.01 as compared with group B.

^#^ *P* < 0.05 as compared with group C.

**Table 5:** The expression of TNF-*α* and IL-6 immunopositive cells of the CCl_4_-induced liver tissue after 8 weeks of resveratrol treatment.

Groups	TNF-*α* (%)	IL-6 (%)
B	28.5 ± 2.4	18.2 ± 0.7
C	15.3 ± 3.6*	12.4 ± 0.3*
D	10.6 ± 4.7**	8.2 ± 0.6**

The expression of TNF-*α*, IL-6 was not detectable in livers of normal control group rats.

Approximately, 200 cells were counted per field, five fields were examined per slide, and five slides were examined per group.

Values are presented as mean ± S.E.M and results were analyzed by One way ANOVA confirmed by Dunnett's test as appropriate using graph pad prism.

*P < 0.05 and **P < 0.01 as compared with Group B.

**Table 6:** Quantification of hepatic apoptosis of rats at the end of the study.

Groups	Apoptotic index^a^ (AI)
B	21.3 ± 1.2
C	13.71 ± 0.17*
D	10.60 ± 0.28**

Group B, CCl_4_-administered rats; group C, resveratrol-(100 mg/kg) treated rats; group D, resveratrol-(200 mg/kg) treated rats. Results are mean ± S.E.M for 10 animals; One way ANOVA followed by Dunnett's multiple comparison test.

*P < 0.05  and **P < 0.01 as compared to group B.

Approximately 200 cells were counted per field, five fields were examined per slide, and five slides were examined per group.

^a^The percentage of TUNEL positive apoptotic cells was denoted as apoptotic index (AI).
